# Acute Toxicity and Neuroprotective Effect of “RJ6601”, a Newly Formulated Instant Soup, in Geriatric Rats

**DOI:** 10.3390/foods14020277

**Published:** 2025-01-16

**Authors:** Rujikarn Chaisanam, Jintanaporn Wattanathorn

**Affiliations:** 1 Department of Physiology and Graduate School (Neuroscience Program), Faculty of Medicine, Khon Kaen University, Khon Kaen 40002, Thailand; rujikan777@gmail.com; 2 Department of Physiology, Faculty of Medicine, Khon Kaen University, Khon Kaen 40002, Thailand; 3 Research Institute for High Human Performance and Health Promotion, Khon Kaen University, Khon Kaen 40002, Thailand

**Keywords:** instant soup, acute toxicity, neuroprotective effect

## Abstract

Given its antioxidant effects and central nervous system benefits, we hypothesized that RJ6601 should improve neurodegeneration in the hippocampus, a region critical for cognition and the maintenance of quality of life (QoL). To assure its safety, a single fixed dose of 2000 mg/kg BW was administered to female Wistar rats (250–450 g, 18 months old) to test the acute toxicity of RJ6601. No mortality and toxicity signs were observed. To prove that RJ6601 can protect against age-related neurodegeneration, RJ6601 at doses of 200 and 400 mg/kg BW was administered to the female Wistar rats once daily for 4 weeks. At the end of the study period, assessments were conducted to evaluate the neuron density; MDA levels; and activities of SOD, CAT, GSH-Px, AChE, total MAO, MAO-A, and MAO-B in the hippocampus. Our results reveal increased neuron density, SOD, CAT, and GSH-Px but decreased MDA, AChE, total MAO, MAO-A, and MAO-B in the hippocampi of female Wistar rats subjected to RJ6601 treatment at both doses used in this study. Therefore, RJ6601 is considered to have low toxicity and may improve neurodegeneration as well as cholinergic and monoaminergic dysfunctions. Subchronic toxicity studies and clinical trials are essential to confirm the safety of RJ6601 consumption and its health benefits.

## 1. Introduction

The global aging population is expanding due to improvements in technology and healthcare systems [1]. It has been reported that the efficiency of physiological functions decreases with age, while the susceptibility to various diseases increases [2]. Accumulative lines of evidence in this decade have demonstrated that a gradual decrease in physiological functions and health span is associated with aging. The oxidative theory of aging suggests that a reduction in oxidative stress, either by reducing the pro-oxidant load, increasing antioxidant defenses, or some combination of both, can increase health span [3]. In addition, oxidative stress also plays a crucial role in age-related pathology, which in turn disturbs the quality of life [4]. Based on the aforementioned crucial roles of oxidative stress, oxidative stress modulation has been focused on as a target for controlling aging and a healthy lifespan [5]. It has been demonstrated that dietary antioxidants, including plant polyphenolic compounds such as flavonoids, can prolong the lifespan by scavenging free radicals and improving oxidative stress status [6]. Furthermore, dietary supplementation with high dietary fiber can also improve oxidative stress by decreasing its accumulation [7]. Interestingly, a recent study demonstrated that mice fed a dietary-fiber-enriched diet for 8 weeks showed improved antioxidant capacity, reduced inflammation, and decreased neuronal cell necrosis in the hippocampus, along with an increase in the expression of *Bifidobacterium* and *Lactobacillus* spp. [8]. Owing to the health benefits of flavonoids and dietary fiber, the anti-aging effects of supplements enriched with these compounds have gained much attention.

It has been reported that the brain is an organ that plays a crucial role in controlling lifespan [9] and quality of life (QoL) [10]. It is one of the most sensitive to age-related changes due to its robust metabolism. The aging brain undergoes reductions in neurotransmitter levels [11] and gray matter volume [12], particularly in the hippocampus, a brain area susceptible to cognitive aging [13] but crucial for maintaining quality of life [14].

It has been reported that the reduction in hippocampal volume follows a non-linear trajectory beginning in middle age. The rates of hippocampal loss in middle age and the elderly are 0.18% and 0.3%, respectively [15]. This change is attributed to neuronal loss and a reduction in neurogenesis [16,17]. This change is also associated with age-related cognitive decline [18]. In addition to the structural change mentioned earlier, neurotransmitter disturbances such as acetylcholine and monoamine transmitters also play a crucial role in cognitive aging [19,20]. Therefore, protection against hippocampal neuronal loss has gained much attention as a target for extending lifespan and slowing down cognitive aging to maintain QoL in adulthood and old age. To the best of our knowledge, no supplement targeting neuroprotection against hippocampal neurodegeneration to prolong the lifespan and maintain QoL in the elderly is currently available, highlighting the need for the development of such a supplement.

Recently, we developed “RJ6601”, a newly formulated instant soup, by substituting wheat flour with banana-derived resistant starch and adding a mixture of kale-derived green colorant and Bael syrup as functional ingredients. It contains 84.42 ± 0.02 µg GAE/g of extract of phenolic compounds. In addition, it also contains gallic acid and marmelosin at concentrations of 5 ± 0.000 and 11 ± 0.00 µg/g of sample powder, respectively. Moreover, RJ6601 is also enriched in dietary fiber (8.42 g/100 g). Our previous study demonstrated that RJ6601 could promote mental wellness through antidepressive, anxiolytic, and cognitive-enhancing effects. It was also shown to protect against neurodegeneration in the frontal cortex [21]. Therefore, the potential of RJ6601 to serve as a supplement targeting neuroprotection against hippocampal degeneration in order to prolong lifespan and QoL in the elderly has been evidenced. Since RJ6601 is categorized as a novel food, toxicity evaluation is essential to assure the safety of consumption of this novel product [22]. Owing to a lack of data regarding toxicity and neuroprotective effects against hippocampal neurodegeneration in middle-aged rats, we aimed to determine acute toxicity and evaluate neuroprotective effects on hippocampal neurodegeneration in these rats. The possible underlying mechanisms were also investigated.

## 2. Materials and Methods

### 2.1. Preparation of “RJ6601” Instant Soup

RJ6601 was formulated as previously mentioned [21]. In brief, wheat flour, unripe-banana-derived resistant starch, Bael fruit syrup, rice bran oil, onion, fish bone stock, potato, unsalted milk, dried fishbones, natural green colorant, and inulin were mixed. In this formulation, the mixture of unripe banana, Bael fruit syrup, and natural green colorant was served as the functional ingredient. A placebo was also formulated with the same process and formular, except no functional ingredient was added. The detailed formulation of RJ6601 and the placebo is provided in Supplementary Material S1. Each serving of RJ660 provides around 4.21 ± 0.01 Kcal/g, whereas a serving of the placebo soup provides 4.35 ± 0.07 Kcal/g. The contents of dietary fiber and polyphenolic compounds in RJ6601 were around 5.59 ± 0.04 g/100 g of sample and 84.42 ± 0.02 mg GAE/g of sample, whereas the mentioned concentrations in the placebo soup were 1.12 ± 0.06 g/100 g of sample and 64.42 ± 0.02 mg GAE/g of sample, respectively. The preparation details have been previously described elsewhere [21]. The fingerprint chromatogram of RJ6601 is provided in Supplementary Material S2.

### 2.2. Animals and Treatment

The experimental animals in this study were female Wistar rats (weighting 250–450 g, 18 months old) from Northeast Laboratory Animal Center, Khon Kaen University, Thailand. They were housed in standard metal cages (5/cage for the acute toxicity study and 4/cage for the neuroprotective study), kept under standard laboratory conditions (temperature at 23 ± 2 °C, with a 12:12 h light/dark cycle), and given free access to standard rodent food and water ad libitum. All the procedures and experimental protocols were approved by the Institutional Animal Ethics Committee of Khon Kaen University (record no. IACUC-KKU-31/64).

#### 2.2.1. Acute Toxicity Test

After one week of acclimatization, the rats (young female nulliparous and nonpregnant rats) were randomly assigned to receive a single administration of placebo or RJ6601 at a dose of 2000 mg/k BW (N = 5/group). The acute toxicity test was performed following the Organization for Economic Co-operation and Development guidelines for chemical testing (OECD 420, 2002) [23]. The fixed-dose test was used to determine the acute toxicity of the RJ6601 soup at a dose of 2000 mg/kg BW. Careful observation for each rat was carried out after the single administration of a novel food supplement at least during the first 30 min and then at 2, 4, 6, 24, and 48 h. The rats were continually observed for 14 days. Observation parameters, including alertness, grooming, tremors, convulsions, salivation, fur, eyes, diarrhea, lethargy, sleep and coma, changes in physical appearance, injury, pain, and signs of illness, were recorded. The body weight and food and water intake were also observed. Rats were sacrificed at the end of the experiment, and blood was collected for the assessment of hematological and blood biochemical values. Furthermore, gross pathology and histopathological changes in visceral organs were also determined.

#### 2.2.2. Study Design for an Evaluation of the Neuroprotective Effect

After a 1-week acclimatization period, the experimental rats were randomly divided into various groups as described below (N = 12/group; 6 rats in each group were studied for histological change).

Group I: placebo group; rats in this group were orally fed placebo soup.

Group II: positive control-treated group; we used donepezil, a neurotrophic drug commonly used for treating dementia [16], as the positive control based on the information that it improves hippocampal and neocortical neurodegeneration [17].

Group III and Group IV: RJ6601 administered at 200 and 400 mg/kg day; rats in these groups were orally administered RJ 6601 at doses of 200 or 400 mg/kg BW.

All substance administrations in this study were performed once daily at the same time for 4 weeks. Both selected doses of RJ6601 in this study were selected because they exerted a beneficial effect in the central nervous system [21]. At the end of study, the rats were sacrificed, and the neuron density; oxidative stress status, including malondialdehyde (MDA) levels; activities of superoxide dismutase (SOD), catalase (CAT), and glutathione peroxidase (GSH-Px); and neurotransmitter changes via the indirect determination of acetylcholinesterase (AChE), total monoamine oxidase (MAO), monoamine oxidase type A (MAO-A), and monoamine oxidase type B (MAO-B) in the hippocampus were determined.

### 2.3. Biochemical Determinations

#### 2.3.1. Oxidative Stress Marker Assessment

The hippocampi (n = 6/group) were removed and prepared as the homogenate by homogenizing brain tissue with 50 volumes of 0.1 M ice-cold phosphate-buffered saline. Then, the resulting solution was centrifuged at 3000× *g* at 4 °C for 15 min. Following centrifugation, the supernatant was harvested and used for bioassays. Oxidative stress markers such as malondialdehyde (MDA), a metabolic product of lipid peroxidation (LPO), and the primary endogenous antioxidant enzymes, including superoxide dismutase (SOD), catalase (CAT), and glutathione peroxidase (GSH-Px), were assessed and served as indicators reflecting the oxidative stress status of the hippocampus.

A thiobarbituric acid (TBA) test was used to assess the MDA level. In brief, hippocampal homogenate at a volume of 50 μL was mixed with a reaction mixture consisting of 50 μL of 8.1% sodium dodecyl sulfate (SDS) (Sigma-Aldrich, St. Louis, MO, USA), 375 μL of 0.8% thiobarbituric acid (TBA) (Sigma-Aldrich, St. Louis, MO, USA), 375 μL of 20% acetic acid (Sigma-Aldrich, St. Louis, MO, USA), and 150 μL of distilled water (DW). Then, the solution was heated at 95 °C in a water bath for 60 min. Following this step, it was cooled with tap water and mixed with 250 μL of distilled water and 1250 μL of an n-butanol and pyridine solution (15:1; Merck, Darmstadt, Germany) and centrifuged at 4000 rpm for 10 min. The upper layer was separated, and its absorbance was determined at 532 nm. A standard calibration curve was prepared from TMP (1,1,3,3-tetramethoxy propane) at concentrations ranging from 0 to 15 μM (Sigma-Aldrich, USA). MDA levels are expressed as ng/mg protein [21].

To monitor SOD activity, 200 μL of a reaction mixture consisting of 57 mM phosphate-buffered solution (KH_2_PO_4_) (Sigma-Aldrich, St. Louis, MO, USA), 0.1 mM EDTA (Sigma-Aldrich, St. Louis, MO, USA), 10 mM cytochrome C (Sigma-Aldrich, USA) solution, 50 μM xanthine (Sigma-Aldrich, USA), and 20 μL of xanthine oxidase (0.90 mU/mL) (Sigma-Aldrich, St. Louis, MO, USA) was mixed with 20 μL of the hippocampal homogenate. Then, absorbance at 415 nm was recorded. SOD enzymes (Sigma-Aldrich, St. Louis, MO, USA) at concentrations of 0–25 units/mL were used for preparation of the standard curve. The results are expressed as units/mg protein [21].

CAT activity was assessed by mixing 10 μL of hippocampal tissue with a reaction mixture consisting of 50 μL of 30 mM hydrogen peroxide (in 50 mM phosphate buffer, pH 7.0) (BDH Chemicals Ltd., London, UK), 25 μL of 4 M H_2_SO_4_ (Sigma-Aldrich, USA), and 150 μL of 5 mM KMnO_4_ (Sigma-Aldrich, St. Louis, MO, USA). Then, absorbance at 490 nm was monitored [24,25].

The assessment of GSH-Px activity was performed by mixing an assay mixture consisting of 10 μL of 1 mM dithiothreitol (DTT) (Sigma-Aldrich, St. Louis, MO, USA) in 6.67 mM potassium phosphate buffer (pH 7), 10 μL of 50 mM glutathione solution (Sigma-Aldrich, St. Louis, MO, USA), and 100 μL of 30% hydrogen peroxide (BDH Chemicals Ltd., UK) and incubating for 5 min. Then, 20 μL of hippocampal tissue and 10 μL of 10 mM 5,5-dithiobis-2-nitrobenzoic acid (DTNB) (Sigma-Aldrich, St. Louis, MO, USA) were added and incubated with shaking for 5 min. Then, absorbance at 412 nm was detected using a microplate reader. A standard calibration curve was prepared using GSH-Px enzymes (Sigma-Aldrich, St. Louis, MO, USA) at concentrations ranging from 0 to 5 units/mL. The results are expressed as units/mg protein [21].

#### 2.3.2. Assessment of the Cholinergic and Monoaminergic Functions

Acetylcholinesterase (AChE) was investigated and served as an indirect indicator reflecting a cholinergic function. In brief, 25 µL of hippocampal tissue was mixed with a reaction mixture composed of 25 µL of 15 mM acetylthiocholine (ATCI), 75 µL of 3 mM DTNB, and 50 µL of 50 mM Tris-HCL, pH 8.0, containing 0.1% bovine serum albumin (BSA). Then, the mixture was incubated at room temperature for 5 min in a 96-well plate. Following this step, 25 µL of 0.22 U·mL^−1^ of AChE was added and incubated for 5 min at room temperature. Absorbance was measured at 415 nm [26]. Acetylcholinesterase (5–1000 µM) was used as a reference standard. The percentage inhibition was calculated using the following equation:

% inhibition = [1 − (A _sample_/A _control_)] × 100,

where A _sample_ is the absorbance of the sample extract and A _control_ is the absorbance of the blank (50% of aqueous methanol in buffer).

The monoaminergic activity was determined by measuring the total monoamine oxidase (MAO), monoamine oxidase type A (MAO-A), and monoamine oxidase type B (MAO-B) activities. For the measurement of MAO, sample dilution at a concentration range of 0.00005 to 5 mg/mL was prepared. An aliquot of tissue sample at a volume of 40 µL was mixed with 120 µL of amino substrate solution (2.5 mM of p-tyramine in potassium phosphate buffer) and 40 µL of chromogenic solution (1 mM of vanillic acid, 0.5 mM of 4-aminoantipyrine, 4 U/mL of peroxidase in potassium phosphate buffer). Then, absorbance at 490 nm was monitored every 3 min over a period of 42 min using a microplate reader (Chromate 4300). Between readings, the plates were incubated at 37 °C. Absorbance readings were performed [27,28].

The assessment of MAO-A and MAO-B activities was performed using a kynuramine determination assay. A reaction mixture consisting of kynuramine (45 and 30 mΜ for MAO-A and MAO-B), a substrate of MAO-A and MAO-B, was dissolved in DMSO (0–10 mg/mL of extract) and mixed with the hippocampal homogenate. Then, 0.0075 mg/mL of MAO enzyme in potassium phosphate buffer (100 mM, pH 7.4) was added and subjected to incubation at 37 °C for 20 min. At the end of the incubation, 400 μL of 2N NaOH and 1000 μL of distilled water were added to stop the reaction. In this study, 4-hydroxyquinoline produced from kynuramine induced by MAO-A and MAO-B was measured via fluorescence at λex = 310 nm and λem = 400 nm. 4-hydroxyquinoline in potassium phosphate buffer (pH 7.4) at a concentrations range of 0.025–2.00 μM was used for preparation of the standard curve. An aliquot of each concentration at a volume of 500 μL was mixed with 400 μL of 2N NaOH and 1000 μL of distilled water before measuring the fluorescence [29].

### 2.4. Histological Procedure and Nissl Staining

The fixative solution containing 4% paraformaldehyde (Sigma-Aldrich, St. Louis, MO, USA) in 0.1 M phosphate buffer with pH 7.4 was perfused transcardially. Then, the brains were infiltrated with a 30% sucrose (Merck, Damstadt, Germany) solution for 48–72 h. Following this step, the brains were cut on a cryostat (Thermo Scientific™ HM 525 Cryostat, Waltham, MA, USA) as the serial sections at a thickness of 10 µm. The sections were placed onto slides coated with 0.3% aqueous gelatin solution containing 0.05% aluminum potassium sulfate (Sigma-Aldrich, St. Louis, MO, USA). The triplicate coronal sections of brains were stained with 0.25% cresyl violet (Sigma-Aldrich, St. Louis, MO, USA), dehydrated using graded alcohols (70, 95, 100% 2×) (RCI LabScan, Bangkok, Thailand), placed in xylene (Merck, Darmstadt, Germany), and mounted using DPX mountant (Merck, Darmstadt, Germany). The representative sections of brain containing dorsal hippocampus were selected for analysis every 3 sections (interval around 30 μm), and an analysis was performed based on the stereotaxic co-ordinates of the hippocampus from Bregma −3.30 to –3.80 mm [30].

The neuron density in the hippocampus was determined using a BH-2 Olympus light microscope (Olympus Life Science, Tokyo, Japan) at 40× magnification. Counting was performed in three adjacent fields, and the mean number was calculated and expressed as the density of neurons per 255 µm^2^.

### 2.5. Statistical Analysis

The data are presented as the mean ± SEM. Statistical analysis was performed using analysis of variance (ANOVA), followed by a Tukey post hoc test. Differences are considered statistically significant when the *p*-value is less than 0.05.

## 3. Results

### 3.1. Acute Toxicity of the Novel Instant Soup RJ6601

**Figure 1:**
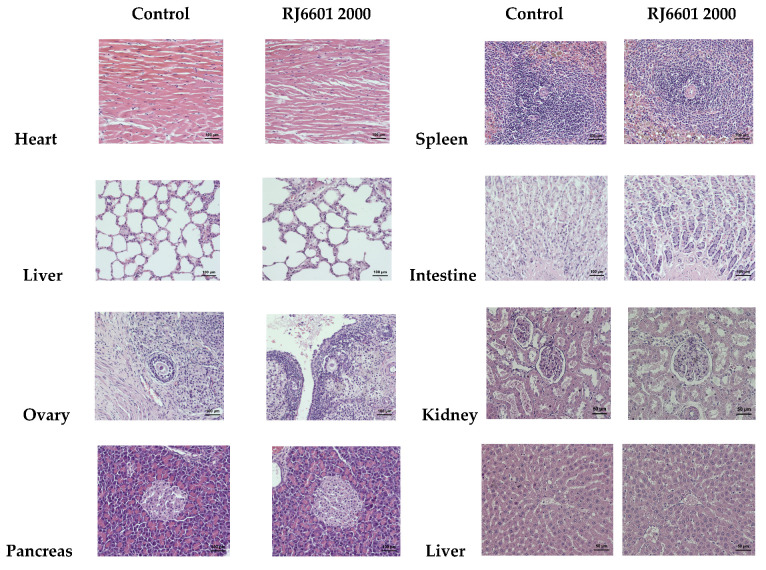
The representative photographs of visceral organs of the placebo group and RJ6601-treated group (2000 mg/kg BW). The visceral organ sections were stained with hematoxylin and eosin and explored under a light microscope at 20× magnification.

The data obtained from this study showed that RJ6601 at a dose of 2000 mg/kg BW did not show any clinical signs of acute toxicity, histopathology of visceral organ changes, hematological and blood biochemical changes, or mortality, as shown in Table 1, Table 2, Table 3 and Table 4 and Figure 1. No significant changes in the consumption of food and water were observed (as shown in Table 5). Therefore, these data indicated that the median lethal dose (LD_50_) of the functional soup should be more than 2000 mg/kg BW.

### 3.2. Changes in Hippocampal Neuron Density

**Figure 2:**
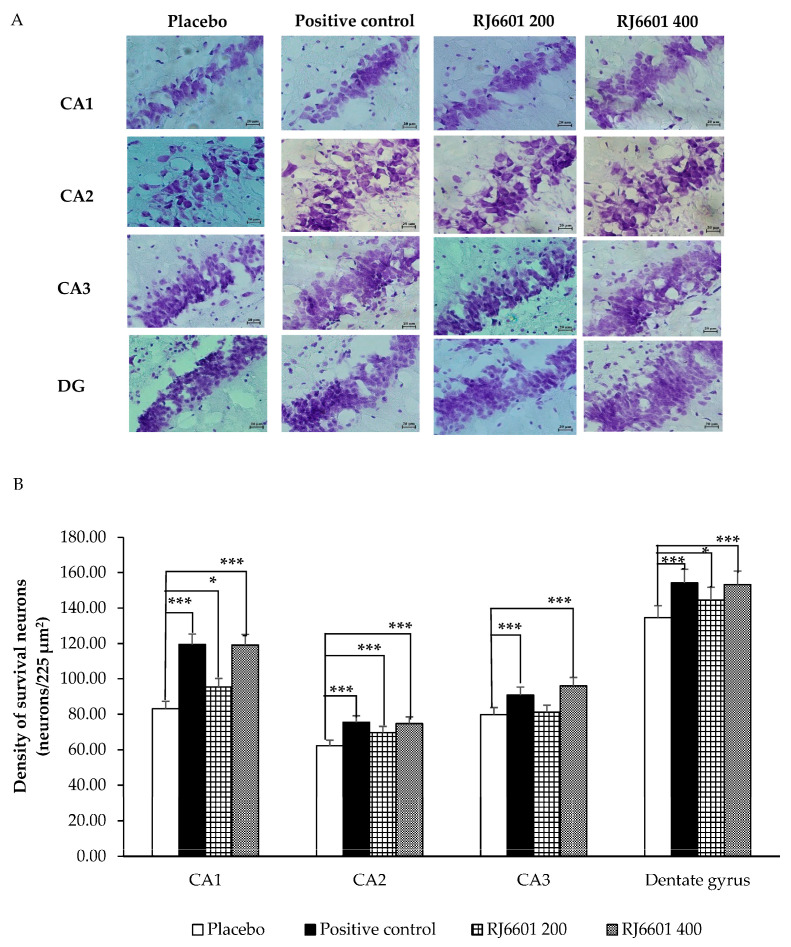
Effect of RJ6601 at doses of 200 and 400 mg/kg BW, positive control, and placebo on density of survival neurons in the hippocampus. (**A**) Representative photographs of CA1, CA2, CA3, and the dentate gyrus under the light microscope. (**B**) Density of survival neurons in CA1, CA2, CA3, and the dentate gyrus (N = 6/group). Data are presented as mean ± SEM. * *p*-value < 0.05 and *** *p*-value < 0.001, compared to placebo group.

Figure 2A,B demonstrate the effect of RJ6601 on the survival neuron density in various subregions of the hippocampus including CA1, CA2, CA3, and the dentate gyrus. It was found that rats that received the positive control or RJ6601 at doses of 200 or 400 mg/kg BW exhibited significantly increased neuron density in CA1 (*p*-value < 0.001, 0.05, and 0.001, respectively, compared to placebo), CA2 (*p*-value < 0.001 all, compared to placebo), and the dentate gyrus (*p*-value < 0.001, 0.05, and 0.001, respectively). In the CA3 subregion, a significant increase in neuron density was observed in rats treated with the positive control or a high dose of RJ6601 (*p*-value < 0.001 all, compared to placebo).

### 3.3. Biochemical Changes

#### 3.3.1. Neurotransmitter Changes

Table 6 demonstrates the effect of RJ6601 on the cholinergic system assessed indirectly via the suppression activity of AChE activity in the hippocampus. When compared to the placebo group, rats treated with a positive control showed a reduction in AChE and total MAO activities in the hippocampus (*p*-value < 0.01 all). Interestingly, RJ6601 at doses of 200 and 400 mg/kg BW significantly suppressed AChE (*p*-value < 0.01, and 0.001, respectively, compared to the placebo group), total MAO (*p*-value < 0.001, all, compared to the placebo group), MAO-A (*p*-value < 0.001, all, compared to placebo group), and MAO-B (*p*-value < 0.01, all, compared to placebo group) in the hippocampus.

#### 3.3.2. Oxidative Stress Changes

Table 7 reveals that the positive control group had significantly decreased MDA levels but increased SOD and CAT activities in the hippocampus (*p*-value < 0.001 all, compared to placebo-treated group). Similarly, RJ6601 at both doses used in this study significantly decreased the MDA level and increased the activities of SOD, CAT, and GSH-Px in the hippocampus (*p*-value < 0.001 all, compared to the placebo group).

## 4. Discussion

The current study shows no mortality, signs of toxicity, or pathology in vital organs following a single administration of RJ6601, a novel instant soup, at a dose of 2000 mg/kg BW. In addition, geriatric rats that received RJ6601 at doses of 200 or 400 mg/kg BW had increased neuron density in CA1, CA2, CA3, and the dentate gyrus of the hippocampus. Furthermore, they also exhibited decreased MDA, AChE, total MAO, MAO-A, and MAO-B but increased SOD, CAT, and GSH-Px activities in the hippocampus.

Soup is healthy and appetizing. Due to today’s fast-paced society and an increasing demand for food that promotes health [31], instant soup has been developed to increase convenience and provide functional benefits. RJJ6601 is a polyphenol- and dietary-fiber-enriched functional instant soup. It is categorized as a novel food because it has been eaten in Thailand for less than 15 years [32]. To assure the safety of its consumption, toxicological study is required [33]. Data obtained from the acute toxicity study of RJ6601 show no mortality and no signs of toxicity up to 2000 mg/kg BW. Therefore, it has been categorized as Group 5 in the toxicity class according to the Globally Harmonized System (GHS) of chemical hazard classification. This suggests that RJ6601 is considered toxicologically irrelevant [34]. Thus, it is worth moving forward to test its efficacy.

Cognitive aging induced by neurodegeneration and neurotransmitter imbalance plays a crucial role in maintaining healthy aging and QoL [16,17,35]; therefore, the hippocampus, which plays a pivotal role in cognitive function [36] and the regulation of the body’s response to stress [37], has been focused on as a supplementary target for rejuvenation. It has been reported that neurodegeneration is a fundamental mechanism of a failing brain preceded by a gradual loss of the essence of being [38]. The neurodegeneration mechanism is attributed to oxidative stress [39,40] and inflammation [41].

Oxidative stress, a condition resulting from the imbalance between the production of ROS and the body’s natural defenses such as antioxidant enzymes, triggers a response to a death signal, leading to apoptosis and necrosis [42]. Under normal circumstances, oxidative stress is counteracted by endogenous antioxidant systems including enzymatic antioxidant enzymes such as SOD, CAT, and GSH-Px [43] and non-enzymatic agents such as melatonin and Coenzyme Q10 [44,45]. Since antioxidant enzymes represent the primary line of defense [43], the current study also explores the effect of RJ6601 on the activities of the enzymes mentioned above. Our results reveal that RJ6601 reduces the MDA level by increasing SOD, CAT, and GSH-Px in the hippocampus. Therefore, the improved oxidative stress status may play a role in the decrease in neurodegeneration, as evidenced by the increase in neuron density in CA1, CA2, CA3, and the dentate gyrus of the hippocampus, giving rise to an improvement in cognitive aging and helping to maintain QoL in the geriatric population.

Beyond neurodegeneration, the reduction in acetylcholine and monoamine transmitters also plays a pivotal role in cognitive aging [46]. The levels of these transmitters depend on the activities of AChE [46] and MAO together with MAO-A and MAO-B [47].

Taken altogether, we suggest that RJ601 improves neurodegeneration in the hippocampus as well as the functions of both cholinergic and monoaminergic systems, which in turn improves cognitive aging and may improve the maintenance of QoL. The limitation of this study is that there is no cognitive measurement. However, our previous work has already revealed that RJ6601 can improve cognitive function in geriatric rats [21]. Therefore, the current study demonstrates that the consumption of RJ6601, a novel instant functional soup, is unlikely to produce toxicity at doses up to 2000 mg/kg BW. It also shows its potential efficacy in protecting against neurodegeneration in brain areas critical for cognitive function and the body’s response to stress, such as the hippocampus. The possible underlying mechanism may involve the improvement in oxidative stress as well as improvement in both cholinergic and monoaminergic functions. In terms of safety and efficacy, RJ6601 shows the potential to serve as a neuroprotectant supplement that can improve cognitive aging and QoL in the geriatric population. However, further research on subchronic toxicity is required to determine the No Adverse Effect Level (NOAEL) or Lowest Observed Adverse Effect Level (LOAEL) of RJ6601 in preclinical and clinical trials, to confirm the safety and benefits of its consumption.

This study does not reveal any dose-dependent effects. The possible explanation may partly be because the observed parameters and the concentration of the soup did not show a linear relationship. Furthermore, raising the concentration of RJ6601 can also increase non-active ingredients, leading to a masking effect. Thus, no dose-dependent effects were detected.

## 5. Conclusions

“RJ6601”, the novel instant functional soup assessed in this study, is unlikely to produce toxic effects due to its high LD50 value (LD50 > 2000 mg/kg BW) and protects against neurodegeneration in the hippocampus, a region involved in cognitive aging and the body’s response to stress in geriatric rats. The mechanisms may be partly associated with improvements in oxidative stress and the modulation of neurotransmitters such as ACh and monoamines. Given the crucial role of cognitive aging in QoL maintenance, we suggest that RJ6601 may serve as a potential supplement for the geriatric population, and it is well suited for modern lifestyles given its convenience. However, preclinical studies on subchronic toxicity and clinical trials in the geriatric population are needed to confirm the safety of consumption, as well as the neuroprotective effects and impact on QoL.

## Figures and Tables

**Table 1:** Toxicity assessment of RJ6601 throughout the 14-day study period (N = 5/group). Data are presented as mean ± SEM.

Direct Observation Parameters	30 min	2 h	4 h	6 h	Day 2–14
Alertness	Normal	Normal	Normal	Normal	Normal
Grooming	Absent	Absent	Absent	Absent	Absent
Hyperactivity	Absent	Absent	Absent	Absent	Absent
Tremors	Absent	Absent	Absent	Absent	Absent
Convulsion	Absent	Absent	Absent	Absent	Absent
Salivation	Normal	Normal	Normal	Normal	Normal
Fur	Normal	Normal	Normal	Normal	Normal
Eyes	Normal	Normal	Normal	Normal	Normal
Diarrhea	Absent	Absent	Absent	Absent	Absent
Lethargy	Absent	Absent	Absent	Absent	Absent
Sleep and coma	Normal	Normal	Normal	Normal	Normal
Injury	Absent	Absent	Absent	Absent	Absent
Pain response	Absent	Absent	Absent	Absent	Absent
Signs of illness	Absent	Absent	Absent	Absent	Absent

**Table 2:** Hematological values of female Wistar rats treated with a single administration of the vehicle and RJ6601 at a dose of 2000 mg/kg BW determined at the end of the 14-day observation period (N = 5/group). Data are presented as mean ± SEM.

Hematological Parameters		Female Wistar Rats	
	Reference Value	Control (Mean ± SEM)	RJ6601 (2000 mg/kg BW) (Mean ± SEM)
Red blood cells (10^6^/μL)	7.16–9.24 (10^6^/μL)	5.84 ± 1.37	7.98 ± 0.25
Hemoglobin (g/dL)	13.7–17.2 (g/dL)	14.57 ± 0.72	15.13 ± 0.43
Hematocrit (%)	38.5–49.2 (%)	45.13 ± 1.49	48.40 ± 1.56
White blood cells (10^3^/μL)	0.96–7.88 (10^3^/μL)	3.02 ± 0.53	2.00 ± 0.20
Platelet count (10^3^/μL)	599–1144 (10^3^/μL)	748.33 ± 12.77	752.33 ± 15.41
Neutrophils (%)	20.1–44.5%	25.70 ± 1.02	29.33 ± 1.14
Lymphocytes (%)	43.7–70.9%	41.43 ± 5.24	68.13 ± 1.19
Monocytes (%)	3.4–9.8%	1.60 ± 0.35	1.37 ± 0.33
Eosinophil (%)	0.7–9.2%	0.80 ± 0.26	1.00 ± 0.23
Basophil (%)	0.0–22.6%	0.27 ± 0.07	0.25 ± 0.00
Mean corpuscular volume (fL)	60–100 (fL)	67.27 ± 2.95	60.63 ± 0.33
Mean corpuscular Hemoglobin (pg)	18–26 (pg)	19.13 ± 0.27	18.97 ± 0.33
Mean corpuscular Hemoglobin concentration (g/dL)	32–36 (g/dL)	31.23 ± 0.59	31.27 ± 0.17
Red blood cell distribution width (%)	12.2 to 16.1 (%)	5.84 ± 1.37	7.98 ± 0.25

**Table 3:** Blood clinical chemistry values of female Wistar rats treated with a single administration of the vehicle and RJ6601 at a dose of 2000 mg/kg BW determined at the end of the 14-day observation period (N = 5/group). Data are presented as mean ± SEM.

		Female Wistar Rats	
Blood Biochemical Parameters	Reference Value	Control (Mean ± SEM)	RJ6601 (2000 mg/kg BW) (Mean ± SEM)
BUN (mg/dL)	7.0–22.0	20.67 ± 0.91	19.47 ± 0.75
Creatinine (mg/dL)	0.50–1.50	0.64 ± 0.03	0.46 ± 0.05
HDL-C (mg/dL)	37–85	58.33 ± 2.85	57.00 ± 0.08
LDL-C (mg/dL)	10–110	5.00 ± 2.00	5.33 ± 2.33
Albumin (g/dL)	3.8–5.4	4.50 ± 0.49	4.27 ± 0.19
Total bilirubin (mg/dL)	0.1–1.2	0.10 ± 0.00	0.10 ± 0.06
Estradiol (pg/dL)	30–400	24.19 ± 3.64	23.10 ± 5.49
ALT (U/L)	7–56	23.00 ± 3.51	39.00 ± 0.07
AST (U/L)	10–36	63.67 ± 2.61	69.00 ± 5.42
LDH (U/L)	140–280	684.67 ± 8.03	687.00 ± 2.58
Uric acid (mg/dL)	2.7–7.0	1.07 ± 0.12	10.3 ± 0.13
Sodium (mmol/L)	135–148	144.00 ± 0.85	142.67 ± 0.88
Potassium (mmol/L)	3.5–5.0	4.53 ± 0.07	4.47 ± 0.18
Bicarbonate (mEq/L)	22.0–26.0	22.63 ± 0.67	24.40 ± 0.06
Chloride (mEq/L)	99–111	97.67 ± 0.88	96.33 ± 0.88
Cholesterol (mg/dL)	127–262	78.67 ± 0.88	75.67 ± 2.32
Protein (g/dL)	6.0–8.3	6.10 ± 0.44	5.97 ± 0.22
Alkaline phosphate (U/L)	75–250	12.33 ± 2.85	11.00 ± 1.15
Triglyceride (mg/dL)	26–145	129.67 ± 4.26	125.67 ± 0.67
T3 (ng/dL)	80–200	86.43 ± 0.97	68.64 ± 2.61
T4 (ng/dL)	4.5–12.5	1.98 ± 0.12	1.73 ± 0.30

**Table 4:** Relative organ weights of female Wistar rats treated with a single administration of the vehicle and RJ6601 at a dose of 2000 mg/kg BW determined at the end of the 14-day observation period (N = 5/group). Data are presented as mean ± SEM.

Relative Organ Weight (%)		Female Wistar Rats	
		Control (Mean ± SEM)	RJ6601 (2000 mg/kg BW) (Mean ± SEM)
Brain		2.15 ± 0.08	2.18 ± 0.02
Salivary gland	Rt.	0.07 ± 0.00	0.06 ± 0.00
	Lt.	0.08 ± 0.00	0.08 ± 0.00
Thymus gland		0.30 ± 0.01	0.35 ± 0.07
Lung		1.41 ± 0.02	1.43 ± 0.08
Heart		0.86 ± 0.3	0.85 ± 0.01
Liver		7.75 ± 0.09	7.89 ± 0.02
Spleen		0.65 ± 0.01	0.68 ± 0.04
Pancreas		1.02 ± 0.07	0.98 ± 0.06
Kidney	Rt.	1.13 ± 0.02	1.13 ± 0.04
	Lt.	1.11 ± 0.04	1.13 ± 0.05
Adrenal gland	Rt.	0.08 ± 0.00	0.05 ± 0.00
	Lt.	0.03 ± 0.00	0.04 ± 0.00
Stomach		1.45 ± 0.13	1.45 ± 1.14
Intestine		4.02 ± 0.15	4.00 ± 0.12
Bladder		0.17 ± 0.00	0.15 ± 0.07
Ovary	Rt.	0.10 ± 0.00	0.10 ± 0.00
	Lt.	0.10 ± 0.00	0.11 ± 0.00

**Table 5:** Relative body weights and food and water intakes of female Wistar rats treated with a single administration of the vehicle and RJ6601 at a dose of 2000 mg/kg BW determined at the end of the 14-day observation period (N = 5/group). Data are presented as mean ± SEM.

Relative Organ Weight (%)	Female Wistar Rats	
	Control (Mean ± SEM)	RJ6601 (2000 mg/kg BW) (Mean ± SEM)
Body weight (g)	404.14 ± 3.02	399.18 ± 5.59
Food intake (g)	11.48 ± 0.23	15.10 ± 0.32
Water intake (mL)	23.63 ± 0.79	27.13 ± 0.21

**Table 6:** The effect of RJ6601 at doses of 200 and 400 mg/kg BW, positive control, and placebo on the activities of acetylcholine esterase (AChE), MAO, MAO-A, and MAO-B in the hippocampus (N = 6/group). Data are presented as mean ± SEM. ** *p*-value < 0.01 and *** *p*-value < 0.001, compared to placebo group.

	Hippocampus			
Groups	AChE Activity	MAO Activity	MAO-A Activity	MAO-B Activity
	(nmol/mg. Protein) (Mean ± SEM)	(µmol/mg. Protein) (Mean ± SEM)	(µmol/mg. Protein) (Mean ± SEM)	(µmol/mg. Protein) (Mean ± SEM)
Placebo	1.41 ± 0.08	0.22 ± 0.01	0.23 ± 0.05	0.23 ± 0.04
Positive control	1.09 ± 0.06 **	0.14 ± 0.02 **	0.17 ± 0.01	0.26 ± 0.02
RJ6601 200	1.08 ± 0.04 **	0.09 ± 0.03 ***	0.07 ± 0.03 ***	0.11 ± 0.02 **
RJ6601 400	0.96 ± 0.07 ***	0.06 ± 0.01 ***	0.05 ± 0.02 ***	0.10 ± 0.01 **

Data are presented as mean ± SEM. ** *p*-value < 0.01 and *** *p*-value < 0.001, compared to placebo group.

**Table 7:** Effect of RJ6601 at doses of 200 and 400 mg/kg BW, positive control, and placebo on the activity of oxidative stress markers MDA, SOD, CAT, and GSH-Px in the hippocampus (N = 6/group). Data are presented as mean ± SEM. *** *p*-value < 0.001; compared to placebo group.

Groups	Hippocampus			
	MDA Levels	SOD Activity	CAT Activity	GSH-Px Activity
	(ng/mg Protein) (Mean ± SEM)	(Units/mg Protein) (Mean ± SEM)	(Units/mg Protein) (Mean ± SEM)	(Units/mg Protein) (Mean ± SEM)
Placebo	1.30 ± 0.03	26.92 ± 0.21	3.40 ± 0.20	3.76 ± 0.20
Positive control	0.41 ± 0.05 ***	41.75 ± 1.65 ***	5.92 ± 0.02 ***	3.85 ± 0.02
RJ6601 200	0.61 ± 0.04 ***	53.62 ± 3.38 ***	6.22 ± 0.40 ***	7.17 ± 0.40 ***
RJ6601 400	0.53 ± 0.03 ***	59.32 ± 2.32 ***	7.10 ± 0.44 ***	8.27 ± 0.44 ***

Data are presented as mean ± SEM. *** *p*-value < 0.001, compared to placebo group.

## Data Availability

The original contributions presented in this study are included in the article/Supplementary Materials. Further inquiries can be directed to the corresponding author.

## Supplementary Materials

The following supporting information can be downloaded at: https://www.mdpi.com/article/10.3390/foods14020277/s1, Supplementary Material S1 shows details of the ingredients used in the placebo and RJ6601. Supplementary Material S2 shows the fingerprint chromatogram of RJ6601.
