# Supplementary material for: Acute Toxicity and Neuroprotective Effect of “RJ6601”, a Newly Formulated Instant Soup, in Geriatric Rats
Source: Foods. 2025 Jan 16;14(2):277. doi: 10.3390/foods14020277 (PMC11765135; doi:10.3390/foods14020277)
Supplement: Supplementary file 1 [file foods-14-00277-s001.zip › Table Supplementary 1 Composition of RJ6601 and placebo soup.pdf]

Table Supplementary 1 Composition of RJ6601 and placebo soup (g/100g).

| **Ingredients** | **Placebo** | **RJ6601** |
|---|---|---|
| Wheat flour | 50.0 | 25.0 |
| Unripe banana-derived resistant starch | 0.0 | 25.0 |
| Bael fruit syrup | - | 4.0 |
| Corn syrup | 4.0 | - |
| Rice bran oil | 1.3 | 1.3 |
| Onion | 12.0 | 12.0 |
| Fish bones stock | - | 20.0 |
| Salt | 0.1 | 0.1 |
| Water | 20.0 | - |
| potato | 3.8 | 3.8 |
| Unsalted milk | 3.2 | 3.2 |
| Dried fishbones | 0.2 | 0.2 |
| Natural green colorant | - | 0.4 |
| Synthetic green colorant | 0.4 | - |
| Inulin | 5 | 5 |
| | 100.0 | 100.0 |
