# Supplementary figures and images for: Acute Toxicity and Neuroprotective Effect of “RJ6601”, a Newly Formulated Instant Soup, in Geriatric Rats
Source: Foods. 2025 Jan 16;14(2):277. doi: 10.3390/foods14020277 (PMC11765135; doi:10.3390/foods14020277)

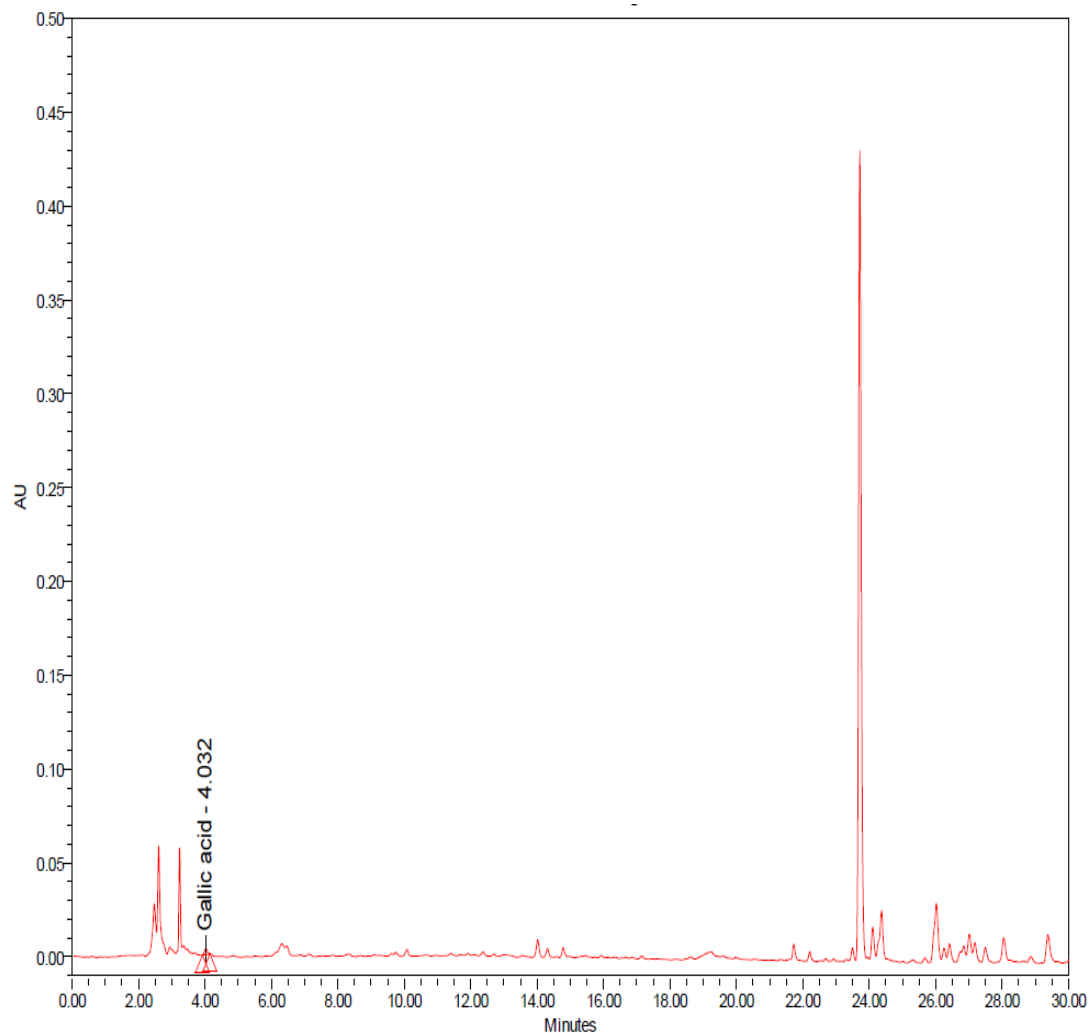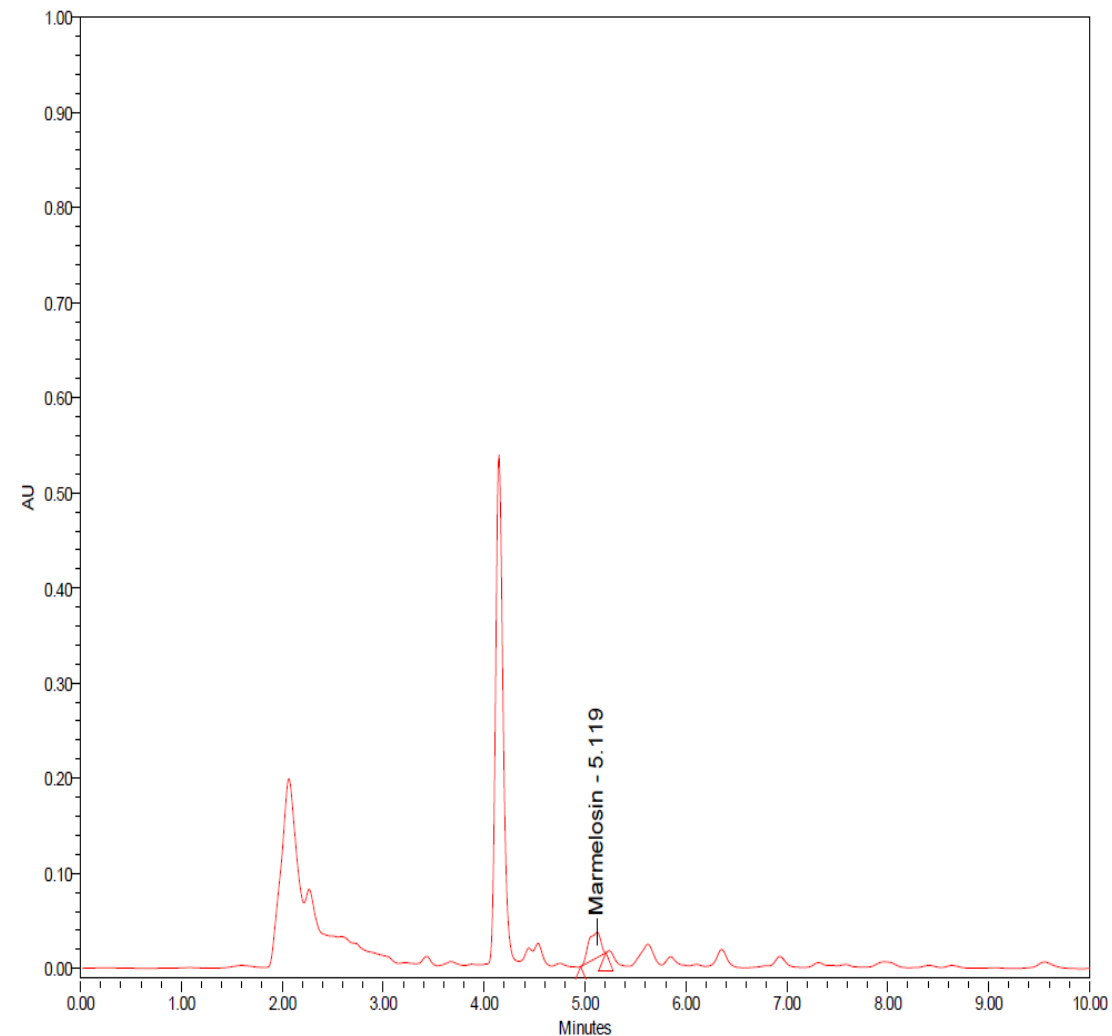

Supplementary material 2 Chromatogram analysis of RJ6601 A)  
Gallic acid content B) Marmelosin

Supplement: Supplementary file 1 [file foods-14-00277-s001.zip › Supplementary 2 Chromatogram.pdf]
